# Some Remarks on Hydrocephalus Internus

**Published:** 1790

**Authors:** Edward Ford

**Affiliations:** Surgeon of the Westminster General Dispensary


VII. *Some Remarks on Hydrocephalus Internus. Communicated in a Letter to Dr.* Simmons, *F. R. S. by Mr.* Edward Ford, *Surgeon of the Weftminfter General Difpenfary.*

IN perufing the defcriptions of hydrocephalus internus, in the writings of Whytt and Fothergill, the reader cannot but remark a ftriking difference in the two accounts with refpect to the duration of the difeafe. The firft of thefe writers (Dr. Whytt) obferves, that children who have water in the ventricles of the brain fhow fymptoms of the difeafe for fome weeks before their death; while Dr. Fothergill, on the other hand, afferts, that, in general,

 his his patients have been feized and taken off in fourteen days, and that he has been feldom able to trace the commencement of it to an earlier period than three weeks before its fatal termination.

This difference may, perhaps, be reconciled in fome meafure by ftating that the hydrocephalus internus is to be traced to various caufes; that it may proceed in fome inftances from injuries to the encephalon from external accidents; or that, in other cafes, the accumulation of fluid may be owing to induration or fchirrous affections of the cerebrum or cerebellum, the lymphatics on the furfaces of thefe parts being, in confequence of fuch morbid affections, unable to perform their function of abforbing the exhaled fluid.

It may be fuggefted alfo, that hydrocephalus internus fometimes arifes in confequence of inflammation affecting the pia mater, and that in this cafe the water is effufed between the membranes of the brain; but if fuch an affection fhould extend to the plexus choroides, or more internal part of the brain, the fluid will then accumulate in the ventricles. In thefe different fituations of the parts, all of which might give occafion to the difeafe, a watery fluid ef-

 fufed fufed within the cranium would fhow its effects upon the conftitution differently, according to its caufe. Thus a fudden accumulation of fluid, from inflammation of the plexus choroides or pia mater, might produce a fatal cataftrophe with the rapidity defcribed by Dr. Fothergill; while an accumulation that fhould proceed more flowly, from fchirrous affections of the cerebellum or cerebrum, would accord with the more tardy progrefs of the difeafe as delineated by Dr. Whytt.

In reafoning on this difeafe it is impoffible to forbear making ufe of the analogy which the dropfies of other cavities of the human body afford; as, for inftance, that of the tunica vaginalis teftis, in which a collection of water may derive its origin from fudden inflammation of the membrane caufed by external injury, or the hernia humoralis: or the accumulation may proceed from venereal, fcrophulous, or fchirrous affections of the teftis or epididymis. All thefe cafes are to be accurately diftinguifhed from each other, and are likewife very different from the difeafe known by the name of the genuine hydrocele.

How far fimilar diftinctions are applicable to the hydrocephalus internus will appear more particularly particularly from the two following cafes, in both of which I had an opportunity of afcertaining the morbid appearances in the brain by diffection:

### CASE I.

On the 31ft of December, 1789, I was defired to examine the body of James Swarfberg, of Great Marlborough Street, who had died the day before of a difeafe which had the appearance of hydrocephalus internus. The fhort narrative I fhall give of the fymptoms previous to his death is taken partly from his mother's report, and partly from my own obfervations, having vifited him occafionally for three months prior to his diffolution.

He was nine years of age, of a dark complexion, had been a very healthy and lively child, had the fmall pox and meafles in his infancy, and in every refpect enjoyed an uninterrupted ftate of good health till within eleven months before his death. The firft fymptom of illnefs was a violent and almoft continual head-ach. He was afterwards obferved to fquint with his left eye; and in walking in the ftreet he frequently ftaggered, and would run to a poft to fupport himfelf, crying out, "Oh!

" my head; I am fo dim I can't fee." In a little time he was unable to walk without ftaggering, and for eight months before his death he was carried out in a woman's arms, not being able to fupport himfelf on his legs. At this time he entirely loft his fight, and his fpeech became very inarticulate. He made attempts to exprefs his wants; but, faultering in his pronunciation, he was fome minutes before he could make himfelf underftood. When I faw him he was quite blind; the pupil of his eyes feemed to be immoveably fixed, being incapable of contraction on being expofed to the ftrongeft light. He was confined to his bed, had no ufe of his arms or legs, uttered a confufed noife on being fpoke to, was much emaciated, complained of pain in his head, and exhibited a dreadful fcene of diftrefs.

During the whole courfe of the difeafe, to its final termination, he ate his food voracioufly, but vomited every day. He was fometimes very coftive, and at other times difcharged his urine and fæces, the latter of which were of a very black colour, involuntarily.

In the beginning of the difeafe he was heavy and drowfy; afterwards he became watchful, and at length comatofe again. For fix weeks before before his death the futures of the cranium began to give way, particularly the futura coronalis, between the indentations of which there was a confiderable vacancy.

On opening the head after death this feparation of the futures was very confpicuous. In the futura coronalis there was an intervening fpace of half an inch between the bones; and in the angle formed by the meeting of the lambdoidal future with the fagittal there was a ftill larger fpace entirely unoccupied by bony fubftance, the occipital bone being quite detached, and moving eafily on the parietal bones.

There was no fluid between the cranium and dura mater. The veffels of the pia mater were rather turgid with blood at the hind part of the head; and in the ventricles of the brain there were twelve ounces of clear lymph not coagulable by a boiling heat.

The plexus choroides was fmall and pale. The communication between the two lateral ventricles was large enough to admit a finger to pafs with facility from one to the other.

No other part of the encephalon exhibited a morbid appearance, till the cerebellum was examined, and this was found to be a very hard difeafed fubftance, fchirrous, and refifting to the the touch, being almoſt as griſtly as a ſchirrous tumour of the breaſt is commonly found to be. The whole of it was of an uniform cineritious colour, very different from that beautiful appearance which reſults from the ramifications of the cortical and medullary portions of the cerebellum, ſo well known by the name of arbor vitæ.

### CASE II.

The ſubject of this caſe was a girl, four years old, who had been a lively healthy child till two months before her death, when ſhe fell from a chair upon the ground. The conſequences of the accident were not much attended to at the time, though ſhe bled at the noſe, was ſick, vomited, and was for a little while ſenſeleſs. She continued, however, after this to play about the houſe, without much inconvenience, for a fortnight, when ſhe became feveriſh and languid, and was confined to her bed. She ſoon after loſt the uſe of her right arm, and the left ſide of her face became convulſed; her eyelids were paralytic for ten days preceding her death; her pulſe frequently intermitted; the pupils of her eyes were dilated; ſhe loſt the power of ſpeaking; and at length became comatoſe and died.

For this account of the progreſs and termination of the diſeaſe I am indebted to Dr. Thynne, who attended the patient during the laſt three weeks of her life, and who omitted nothing that humanity or ſkill could dictate for her preſervation.

On examination of the head after death, I found the cranium rather protruding at the os frontis and oſſa parietalia. The pericranium adhered firmly to the ſkull; the indentations of the ſutures were cloſe; there was no fluid between the ſkull and dura mater, or between the membranes; the veſſels of the pia mater were not loaded with blood; nor were the ſinuſes remarkably full.

The ventricles were found to contain eight ounces of water. The plexus choroides was ſmall and pale. In the right ventricle an hydatid of the ſize of a ſmall cheſnut was found attached to the plexus choroides.

No other morbid appearance was perceptible till the brain was removed from the baſis of the ſkull, when the Pons Varolii was found to be changed to a ſchirrous, diſeaſed ſubſtance; the glandula pituitaria diminiſhed and altered from from its natural appearance ; and the bafis of the fkull, on which the cerebellum lays, feemed to be lefs fmooth than ufual, the dura mater not clofely adhering to it in this part.

The reflections to be drawn from thefe diffections, were they to be confidered merely as cafes of hydrocephalus, would lead to very different conclufions from thofe fuggefted by Dr. Fothergill, who confiders the hydrocephalus internus as a difeafe that can rarely be traced in its origin above three weeks *, and cautions the practitioner from looking back to diftant æras for the foundation of a malady with which they have no connexion ; yet afterwards † he fays that he does not defpair of the beginning of the difeafe being traced a little higher, of its caufes being better afcertained, and its character fixed with more precifion.

Dr. Whytt, on the contrary, thinks the difeafe may, in fome inftances, be traced to a diftant origin, and produces a cafe where he thinks it probable that the origin may be traced ten months back to the meafles, which firft broke the health of the child. He alfo men-

* Medical Obfervations and Inquiries, Vol. IV. page 44.

† Ibid. page 54.

tions tions a case of hydrocephalus in which the Thalami nervorum opticorum were much diseased.

M. Petit furnishes instances of the glandula pituitaria being often scirrhous in this disease. He also mentions the circumstance of the sutures being separated; which symptom Dr. Whytt says is very unlikely to take place in a child above two years old *.

The valuable records of Bonetus supply us with numerous observations of large collections of fluid in the ventricles, attended with diseases of various parts of the brain. The symptoms of the malady had been observable in some cases for a long time; in one case, that of an adult, for eighteen months.

I hope it will not be thought too rash a conclusion from such great authorities as I have mentioned, and others might be adduced, to infer, that the hydrocephalus internus is, in many cases, not to be considered as a disease *sui generis*, or that it is of itself the sole cause of the mischief which ensues to the constitution, but that it is often the effect proceeding

* This symptom was a very prominent mark of the disease in the first case related in this paper.

from thofe difeafes which it has been faid to produce.

There are few, I believe, who will conceive that the accumulation of water in the ventricles will produce fcirrhus of the Thalami nervorum opticorum, or of the cerebellum; but it is very eafy to know that an accumulation of fluid may enfue, if thofe parts are fo difeafed that the lymphatics on their furface cannot exercife their function.

That thefe parts may become difeafed in confequence of the fmall pox, meafles, fcrophula, or other remote caufes, as well as the mefenteric glands, the lymphatic glands of the neck, or thofe of the lungs, is very likely to happen; and in fuch cafes the remote caufe muft be inveftigated whenever the means of cure are taken into confideration.

The fymptoms of hydrocephalus internus undoubtedly come on with great rapidity in fome cafes, without any obvious predifponent caufe. I have feen the difeafe produced more than once by external injury; from a blow upon the head the fkull has been fractured *, and on opening the integuments there has been a-

* See Vol. VIII. of this work, pages 417 and 421.

drain, drain, for a confiderable time, of clear limpid fluid from the infide of the fkull difcharged through the fracture.

I had fome time ago a melancholy inftance of the difeafe in a child of two years old, and apparently occafioned by the careleffnefs of a nurfe who had ftruck its head forcibly againft the bottom of a bathing tub: comatofe fymptoms came on quickly, and the child died in ten days. I examined the head, but found no other morbid appearance than three ounces of water in the lateral ventricles.

*Golden Square,*
February 19, 1790.

